# SENSE OF PURPOSE IN LIFE AND REDUCED LIKELIHOOD OF FUTURE DRUG MISUSE

**DOI:** 10.1093/geroni/igz038.1258

**Published:** 2019-11-08

**Authors:** Eric S Kim, Carol Ryff, Afton Hassett, Chad Brummett, Charlotte Yeh, Victor Strecher

**Affiliations:** 1 Harvard T.H. Chan School of Public Health, Boston, United States; 2 University of Wisconsin, Madison, Wisconsin, United States; 3 University of Michigan, Ann Arbor, Michigan, United States; 4 AARP Services, Inc., Washington, District of Columbia, United States

## Abstract

A stronger sense of purpose in life is hypothesized to reduce the likelihood of drug misuse because it has been linked with several protective factors including: increased ability to handle stress and pain tolerance, decreased impulsivity, and reduced risk of depression and chronic conditions. However, the association between purpose in life and drug misuse has been understudied. We tested whether people with a stronger sense of purpose in life had a decreased likelihood of incident drug misuse 9-10 years later. We also tested whether people with a stronger sense of purpose were less likely to cope with stress by misusing drugs. Participants were drawn from the Midlife in the United States Study (MIDUS; n=3,483) and from a stress coping module of the Health and Retirement Study (HRS; n=498). Among MIDUS respondents not misusing drugs at baseline, people in the highest quartile of purpose (compared to people in the lowest quartile) had 42% reduced odds (95% CI: 0.37-0.92) of incident drug misuse 9-10 years later in the fully-adjusted model (e.g., sociodemographic factors, depression, chronic conditions, and chronic pain). Among HRS respondents, people in the highest purpose quartile had 65% decreased odds (95% CI: 0.14-0.89) of misusing drugs to cope with stress in the fully-adjusted model. A growing number of intervention studies show that purpose in life can be raised. With additional research, these data suggest that sensitively tailored and administered purpose in life may reduce the likelihood of drug misuse and help stem the tide of our nation’s growing drug epidemic.

